# Polymorphism in alpha-synuclein oligomers and its implications in toxicity under disease conditions

**DOI:** 10.3389/fmolb.2022.959425

**Published:** 2022-08-12

**Authors:** Je Min Yoo, Yuxi Lin, Yunseok Heo, Young-Ho Lee

**Affiliations:** ^1^ BioGraphene Inc, Los Angeles, CA, United States; ^2^ Research Center for Bioconvergence Analysis, Korea Basic Science Institute, Ochang, South Korea; ^3^ Department of Bio-Analytical Science, University of Science and Technology, Daejeon, South Korea; ^4^ Graduate School of Analytical Science and Technology, Chungnam National University, Daejeon, South Korea; ^5^ Research Headquarters, Korea Brain Research Institute, Daegu, South Korea

**Keywords:** alpha-synuclein, oligomers, polymorphism, fibrillation, toxicity, disease progression, Parkinson’s disease

## Abstract

The major hallmark of Parkinson’s disease (PD) is represented by the formation of pathological protein plaques largely consisting of α-synuclein (αSN) amyloid fibrils. Nevertheless, the implications of αSN oligomers in neuronal impairments and disease progression are more importantly highlighted than mature fibrils, as they provoke more detrimental damages in neuronal cells and thereby exacerbate α-synucleinopathy. Interestingly, although generation of oligomeric species under disease conditions is likely correlated to cytotoxicity and different cellular damages, αSN oligomers manifest varying toxicity profiles dependent on the specific environments as well as the shapes and conformations the oligomers adopt. As such, this minireview discusses polymorphism in αSN oligomers and the association of the underlying heterogeneity in regard to toxicity under pathological conditions.

## 1 Introduction

The pathogenesis of Parkinson’s disease (PD), the second most common type of neurodegenerative disease, is multifactorial with various known molecular and transgenic factors. Irrespective of the precise cause, α-synucleinopathy is considered as the major pathological hallmark of PD characterized by the abnormal fibrillation and subsequent aggregation of a presynaptic neuronal protein called α- synuclein (αSN) (Spillantini et al., 1997; Dawson and Dawson, 2003). Although different putative functions of αSN such as the regulation of neurotransmitter release have been suggested, its exact functions under normal conditions remain elusive (Stefanis, 2012). The implications of αSN most prominently manifest in different neurodegenerative diseases including PD, as pathological deposits called Lewy bodies (LBs) are largely formed by αSN amyloid fibrils (Kim et al., 2014). As the disease progresses, these deleterious αSN aggregates undergo neuron-to-neuron propagation along the midbrain, which progressively kill off dopamine producing neurons in the substantia nigra (Desplats et al., 2009). Thus, in the early days of studies, researchers devoted their attention to the exploration of mature fibrils in the hopes of understanding the formation of LBs and their association with the disease progression.

Around the late 1990s, however, attention shifted to soluble oligomers, as growing evidence from cellular toxicity studies suggested that oligomeric species provoke more severe detriments to neurons when compared against fibrillar aggregates (Bemporad and Chiti, 2012; Stefani, 2012). The ensuing findings from established *in vitro* and *in vivo* experiments, coupled with the biofluids and tissue samples from human patients, further validate the implications of oligomeric species in triggering and aggravating α-synucleinopathy in PD (Kalia et al., 2013; Cremades et al., 2017). The oligomerization of αSN unavoidably produces a heterogenous population of oligomers varying in size and morphology. Namely, assorted oligomers ranging from small (∼2–5-mers), medium (∼5–15-mers), to large (∼15–150-mers) species can be formed in different shapes (Oliveri, 2019). In addition to the distinctive toxicity profiles these oligomers exhibit, their structural and conformational heterogeneity consequentially gives rise to polymorphism in mature fibrils, which remains crucial for garnering further insight into the formation of LBs (Stefani, 2012). Therefore, beyond their overarching impact in neuronal impairment, understanding polymorphism in αSN oligomers, including drawing a clearer connection between heterogeneity and toxicity, is crucial for a more complete comprehension of disease progression and drug development to yield an effective target selection for PD.

As such, this minireview highlights the importance of polymorphism in αSN oligomers through the correlation with various forms of cellular damages in neurodegenerative diseases, focused on PD. While understanding the *in vivo* formation and progression of αSN oligomers stands as a critical element in drug development, obtaining granular information around the fibrillation process inevitably demands extensive *in vitro* studies to investigate key aspects that are comparably infeasible *in vivo*. Hence, this minireview predominantly focuses on various *in vitro* studies with suggested relevance and correlation to *in vivo* environments, as well as further translation of these findings into clinical settings. Foremost, we discuss the reported pathways of oligomer generation, which primarily take place during the formation and disaggregation of amyloid fibrils. Several noticeable features are highlighted and common across these oligomers, such as size, shape, post-translational modifications, as well as relevance in their distinct toxicity profiles. Based on these unique characteristics, the specific types of damages different oligomeric species provoke in neuronal cells are outlined. Finally, we present several notable modulators for varying types of αSN oligomers, which provide important and timely insight into the ongoing and forthcoming drug development efforts to surmount α-synucleinopathy which exhibits a polymorphic fibrillation process.

## 2 Oligomer polymorphism, toxicity, and disease progression

### 2.1 Generation of oligomers

#### 2.1.1 Amyloid fibril formation

The formation of diverse non-fibrillar aggregates can be identified with several standardized biophysical methods including electron microscope (EM), atomic force microscope (AFM), and fluorescence spectrometer at the onset of amyloidogenesis. Namely, the early stages of αSN fibrillation generate an array of distinct oligomeric species varying in size, shape, stability, and β -sheet content, particularly during the lag phase (Cremades et al., 2017). This is mainly because i) the nucleation and growth of fibrils occurs within a heterogeneous population of small, medium, and large oligomers and ii) even the oligomerization of identical monomers acquires distinct conformations dependent on the specific and unique conditions. These oligomeric species can be either on-pathway intermediates or off-pathway dead end products, however most of them are considered unstable, transient intermediates arising in the path of forming mature fibrils (Stefani, 2012; Karamanos et al., 2019). Although the major difference between on- and off-pathway oligomers is distinguished by the eventual formation of fibrillar assemblies, some researchers only classify the oligomers whose blockage can lead to an apparent prevention of fibril formation as on-pathway oligomers (Dear et al., 2020). It should be noted that the formation of stable off-pathway intermediates by compounds such as polyphenols has been suggested as a strategy to reduce relevant toxicity. However, both on- and off-pathway oligomers are implicated with toxicity in different contexts, depending on many complicated factors, some of which are described in later sections.

#### 2.1.2 Amyloid fibril disaggregation

Previous findings have also demonstrated that mature αSN amyloid fibrils release soluble polymorphic dimers and oligomers, which may provoke severe toxic outcomes to neurons in the vicinity (Cremades et al., 2012; Cascella et al., 2021). Interestingly, oligomers produced from short fibrils elicit significantly more deleterious effects in neurons when compared to species released from long fibrils. This is partly because the release of toxic oligomers occurs from the fibrillar ends; shorter fibrils facilitate a faster release owing to their higher proportion of ends (Cascella et al., 2021). In addition to immediate functional impairments in the neurons, these oligomeric species can be internalized and contribute to progressive diffusion of α-synucleinopathy through neuron-to-neuron transmission. Studies on denatured mature fibrils under supercooling conditions show annular and spherical oligomeric species, which reportedly exhibit similar toxicity levels to oligomers generated during amyloid formation (Kim et al., 2008).

#### 2.1.3 Binding to lipid membranes

The interactions between αSN monomers and the cell membrane are particularly critical during the initial stages of amyloid formation (Terakawa et al., 2018a; Terakawa et al., 2018b; Lin et al., 2022). Upon binding to lipid membranes, αSN monomers oligomerize, primarily to dimers and trimers, as the cross-linking between αSN monomers act to stabilize the conformations of membrane-bound αSN (Cole et al., 2002; Ding et al., 2002). Notably, the membrane-induced oligomerization of αSN may result in the formation of nucleation sites for subsequent aggregation and aggravated α-synucleinopathy. Some researchers suggest that a longer incubation of membrane-bound spherical oligomers results in structural conversion into membrane-bound annular oligomers, where such alteration may contribute to increased toxicity and accelerated disease progression (Ding et al., 2002).

### 2.2 Structure-toxicity relationships

Unlike mature amyloid fibrils, αSN oligomers are predominantly localized in the presynaptic terminals, where they exert harmful impacts on synapses and dendrites. Similar to their provocation of distinct fibrillation kinetics to mature amyloids, differently structured and shaped oligomers elicit distinctive toxic outcomes.

#### 2.2.1 Spherical oligomers

The early onset of the fibrillation process typically produces small spherical oligomers, which further assemble into annular protofibrils or even mature fibrils in the presence of excess αSN monomers (Cremades et al., 2017). While the mechanisms underlying the toxicity of αSN oligomers primarily pertain to various forms of cell membrane perturbation, the interactions between spherical oligomers and the membrane are not particularly pronounced (Surguchov et al., 2017). Therefore, compared to annular oligomers, spherical or globular oligomers are considered more stable and thus generally display less deleterious toxicity profiles (Cremades et al., 2012). In support of this notion, studies on brain tissue samples of multiple system atrophy (MSA) patients manifesting α-synucleinopathy revealed that mild detergent treatment breaks apart the inclusions into 30–50 nm-sized annular oligomers. On the other hand, the same treatment on recombinant wild type (WT) αSN results in the release of spherical oligomers (Pountney et al., 2005). The findings suggest that pathological conditions preferentially form annular oligomers with higher toxicity. However, it should be noted that unlike annular oligomers, spherical oligomers can be internalized by neuronal cells and function as a seed for consequential nucleation and elongation (Danzer et al., 2007). Taken together, although the direct toxicity of spherical oligomers is deemed relatively subtle in eliciting destructive outcomes, their spontaneous conversion to annular oligomers and/or the creation of further nucleation sites may contribute to exacerbated α-synucleinopathy.

#### 2.2.2 Annular oligomers

Annular species have become the focal point for understanding the neuronal impairments induced by αSN oligomers under disease conditions. In addition to the studies with MSA patients’ brain tissue samples showing αSN inclusions are predominantly formed by annular species, several subsequent studies further validate the implications of annular oligomers in toxicity including the specific types of damages they provoke.

##### 2.2.2.1 Membrane damage

In the past, the prevailing hypothesis around the toxicity of αSN oligomers proposed the embedment of annular species into lipid bilayers, which leads to the formation of pore-like protein channels (Kostka et al., 2008). However, further analyses have since corroborated that the intercalation of the oligomers between tightly packed lipid domains induces disintegration of the hydrophobic core where destabilized membrane permeability thus triggers an aberrant transport of molecules across the membrane (Stöckl et al., 2013). Indeed, the updated notion with decreased lipid order is consistent with the observations of elevated lipid flip-flops induced by oligomers. Importantly, membrane destabilization can lead to dysregulation of intracellular calcium homeostasis; several hypotheses consider atypically increased intracellular calcium levels as an important factor contributing to neurodegeneration (Zaichick et al., 2017). In addition to the formation of toxic annular oligomers of 70–90 nm in diameter when bound to the C-terminal of αSN, elevated intracellular calcium levels via unregulated transport of extracellular calcium induce a remarkable increase in the activation of caspase-3 for consequential apoptosis (Danzer et al., 2007). It should also be noted that aberrant ion flux correlates with abnormal patterns in the neuronal excitabilities, which allude to another aspect of neuronal vulnerability in PD.

##### 2.2.2.2 Other damages

**FIGURE 1:**
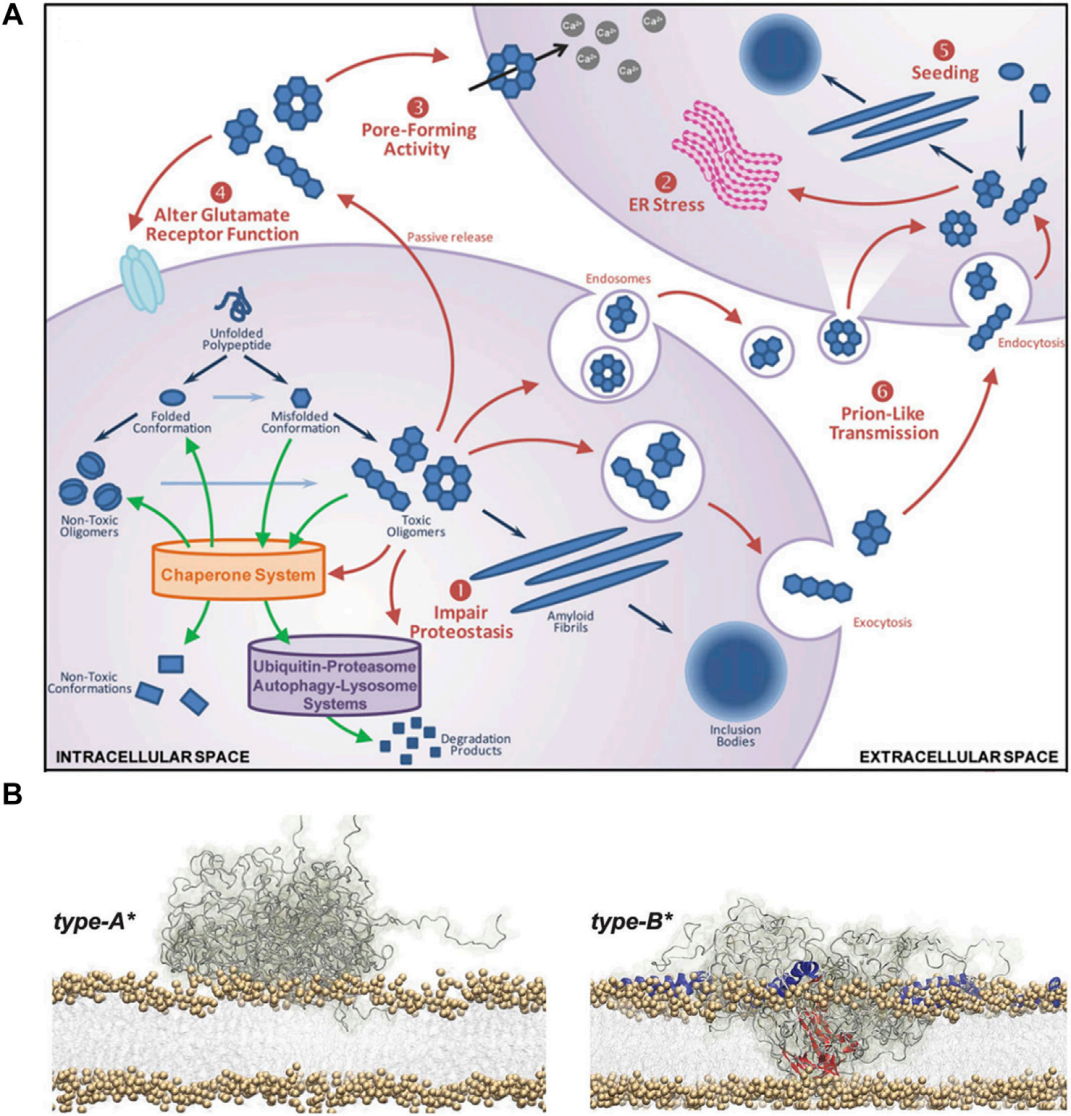
Toxic effects of αSN oligomers. **(A)** Schematic representation of the αSN oligomerization and ensuing toxicity. The outlined process includes: 1) impaired proteostasis, 2) prolonged ER stress, 3) aberrant pore formation, 4) dysfunctional glutamate receptor, and 5) intracellular uptake and seeding followed by 6) neuron-to-neuron transmission of pathological aggregates (Kalia et al., 2013). **(B)** Schematic representation of the membrane destabilization process by two types of oligomers with distinct secondary structure contents. Type-A* oligomers are predominantly unstructured and can only bind to the surface of biological membranes (left). Type-B* oligomers exhibit both disordered (gray) and β-sheet (red) regions where the β-sheet regions penetrate through the lipid bilayers, provoking destabilization (right). In addition, the folding of the N-terminal regions into α-helices (blue) provide more binding regions to the membrane (Fusco et al., 2017).

While growing evidence supports disruption of the plasma membrane integrity via oligomer-membrane interaction, various other forms of cellular damages by αSN oligomers have been reported. Akin to the plasma membrane destabilization, destructive permeabilization into the membranes of mitochondria, endoplasmic reticulum (ER), and various trafficking vesicles can be more readily provoked by annular oligomers. Such damages trigger enhanced oxidative stress, membrane depolarization, and various forms of dysfunction including disruption of the electron transport chain in mitochondria (Stefani, 2012). Impairments in the ER-Golgi membranes are linked to ubiquitin/proteasome system-mediated clearance pathways as well as obstruction of trafficking. In addition, severe lysosomal leakages and/or adverse cytoskeletal changes can manifest (Oliveri, 2019). Finally, membrane-bound forms or internalized soluble species can act as nucleation sites to spread and aggravate α-synucleinopathy (Cascella et al., 2021). Possible toxicity pathways induced by αSN oligomers are outlined in Figure 1A.

#### 2.2.3 Secondary structure

In addition to morphological characteristics, distinct secondary structure contents within similarly shaped and sized αSN oligomers can lead to conflicting outcomes in cytotoxicity. Namely, oligomeric species with richer β -sheet content are likely inclined to make deleterious interactions with cellular membranes as binding is facilitated by increased hydrophobicity (Alam et al., 2019). Fusco et al. employed two different types of spherical oligomers within a similar size range that display contradicting toxicity profiles (Fusco et al., 2017). While type A oligomers did not elicit any disruption in synthetic and cellular membranes, type B oligomers exhibited clear signs of destructive perturbation in the membranes as well as damages from impaired mitochondrial functions and increased ROS levels. Such disparity in toxicity can be attributed to the difference in their secondary structure contents. As shown in Figure 1B, While the type A oligomers predominantly manifest unstructured conformation, the type B oligomers show evident β -sheet and random-coil contents. In addition, unlike the type A oligomers, the type B oligomers display dynamic N-terminal regions, which adopt amphipathic α-helices upon interacting with cellular membranes. Collectively, beyond the type B oligomers’ β -sheet structure that inserts into the membranes and thereby destabilizes integrity, the helical folding of the N-terminal regions further contributes to conspicuous membrane disruption. In support of these findings, Xu et al*.* performed a comparative toxicity analysis of different variants of αSN oligomers (G51D, A30P, E46K, H50Q, and A53T) associated with the onset of familial PD (Xu et al., 2022). Despite their comparable size, overall morphology, and β -sheet content, only G51D oligomers show markedly higher toxicity than WT oligomers. It is fascinating to note that G51D oligomers reveal polymorphism in the α-helical content, where their richer presence results in more detrimental outcomes. Helical folding of the N-terminal part also shows the importance in binding to the membranes of presynaptic vesicle and mitochondria (Terakawa et al., 2018a; Terakawa et al., 2018b; Lin et al., 2022). Thus, besides hydrophobicity and/or the level of β -sheet content, α-helical content stands as an important factor in understanding the structure-toxicity relationships of αSN oligomers.

#### 2.2.4 Post-translational modifications

αSN can undergo several types of post-translational modifications that alter the propensity for aggregation, which thereby affect the toxicity profiles and levels of oligomers (Alam et al., 2019).

##### 2.2.4.1 Phosphorylation

Phosphorylation of αSN plays a critical role in modulating the fibrillation process and relevant neurotoxicity (Barrett and Greenamyre, 2015). While αSN can be phosphorylated at different residues including tyrosine 133 and 136 (Y133 & 136), phosphorylation at serine 129 (S129) is one of the most representative hallmarks of the fibrillation process, which increases the levels of toxic αSN oligomers (Fujiwara et al., 2002). On the other hand, phosphorylation at other residues such as S87 and Y125 can decrease the production of αSN oligomers by modulating the interactions between αSN and the cell membrane (Chen et al., 2009; Paleologou et al., 2010).

##### 2.2.4.2 Nitration

Various reactive nitrogen species (RNS) including peroxynitrite (ONOO^−^) can nitrate tyrosine residues in αSN, which can be detected with 3-nitrotyrosine antibodies. Nitration of αSN may render a protective factor against α-synucleinopathy as *in vitro* nitrated αSN cannot undergo fibrillation while also preventing the fibrillation of non-nitrated αSN in the vicinity (Yamin et al., 2003; Hodara et al., 2004). This can be attributed to the preferential production of stable spherical oligomers that are predominantly octamers with some dimeric and trimeric populations. On the other hand, several contrasting results report that nitrated αSN oligomers’ reduced association with lipid vesicles favors self-aggregation, which promotes the formation of inclusions to exacerbate α-synucleinopathy (Liu et al., 2011). However, it should be noted that the cytotoxicity of nitrated αSN has a deeper connection with the integrin- inducible NO synthase (iNOS)/-focal adhesion kinase (FAK) signaling pathway than with the formation of cytotoxic oligomers.

#### 2.2.5 Other modifications

##### 2.2.5.1 Lipid peroxidation

Under pathological conditions, increased production of reactive oxygen species (ROS) causes lipid peroxidation of various polyunsaturated fatty acids (PUFAs) in the brain that alter αSN fibrillation. Foremost, docosahexaenoic acid (DHA), which localizes at synapses, is known to promote αSN aggregation with the production of heterogeneous oligomers dependent on the molar ratio (De Franceschi et al., 2011; Fecchio et al., 2013). More importantly, peroxidation of DHA is implicated in the generation of reactive aldehydes such as 4-hydroxyl-2-nonenal (4-HNE), when other peroxidation adducts produce acrolein, malondialdehyde (MDA), and 4-oxo-2-nonenal (4-ONE), which all exhibit considerable cytotoxicity by themselves (Esterbauer et al., 1991; Lee and Blair, 2000). Noticeably, covalent modifications of αSN by 4-ONE and 4-HNE are known to trigger the production of highly toxic off-pathway αSN oligomers rich in β -sheet content (Nasstrom et al., 2011). The oligomers induced by 4-ONE are mostly amorphous with higher stability and lower protease-resistance, whereas 4-HNE induces the generation of distinctly shaped species ranging from spherical to annular. This is supposedly due to 4-ONE’s carbonyl group resulting in more potent cross-linking. It should also be noted that increased levels of adducts associated with 4-HNE and acrolein have been found in PD brain tissues (Castellani et al., 2002).

##### 2.2.5.2 Metal ion binding

While the brain maintains proper metal homeostasis, dysregulated levels of metal ions under pathological conditions lead to increased ROS production and aberrant interactions with αSN that stimulate aggregation. In addition to the aforementioned role of Ca^2+^ ions in producing annular oligomers, the implications of different metal ions including Cu^2+^, Fe^3+^, Al^3+^, and Cd^2+^ in promoting the formation of αSN oligomers and fibrils have been highlighted (Uversky et al., 2001). Akin to Ca^2+^, Cu^2+^ also accelerates oligomerization by binding to the C-terminal of αSN, where the presence of Cu^2+^ chelators reposition αSN towards the membrane with decreased aggregation (Wang et al., 2010). It is also important to note that Fe^3+^ results in the creation of destructive ion-permeable pores by inducing the formation of sodium dodecyl sulfate (SDS)-resistant oligomers (Kostka et al., 2008).

##### 2.2.5.3 Nucleic acids

The interplay between αSN and nucleic acids has also been investigated under different conditions. Particularly, double-stranded DNA (dsDNA) interacts with αSN to promote fibrillation along with evident association with the mature fibrils (Cherny et al., 2004; Di Domizio et al., 2012). Namely, soluble αSN oligomers display preferential binding with nucleic acids and glycosaminoglycans (GAG) to accelerate the formation of mature fibrils. Interestingly, such binding decreases the cytotoxicity presumably due to the structural conversion of strongly toxic oligomers into less toxic fibrils (Di Domizio et al., 2012). Nevertheless, the *in vivo* and clinical significance of nucleic acids in αSN fibrillation remains questionable as a recent study demonstrated that amyloid αS binding to dsDNA is weak and nonspecific (Jos et al., 2021).

### 2.3 Modulators targeting oligomers

**FIGURE 2:**
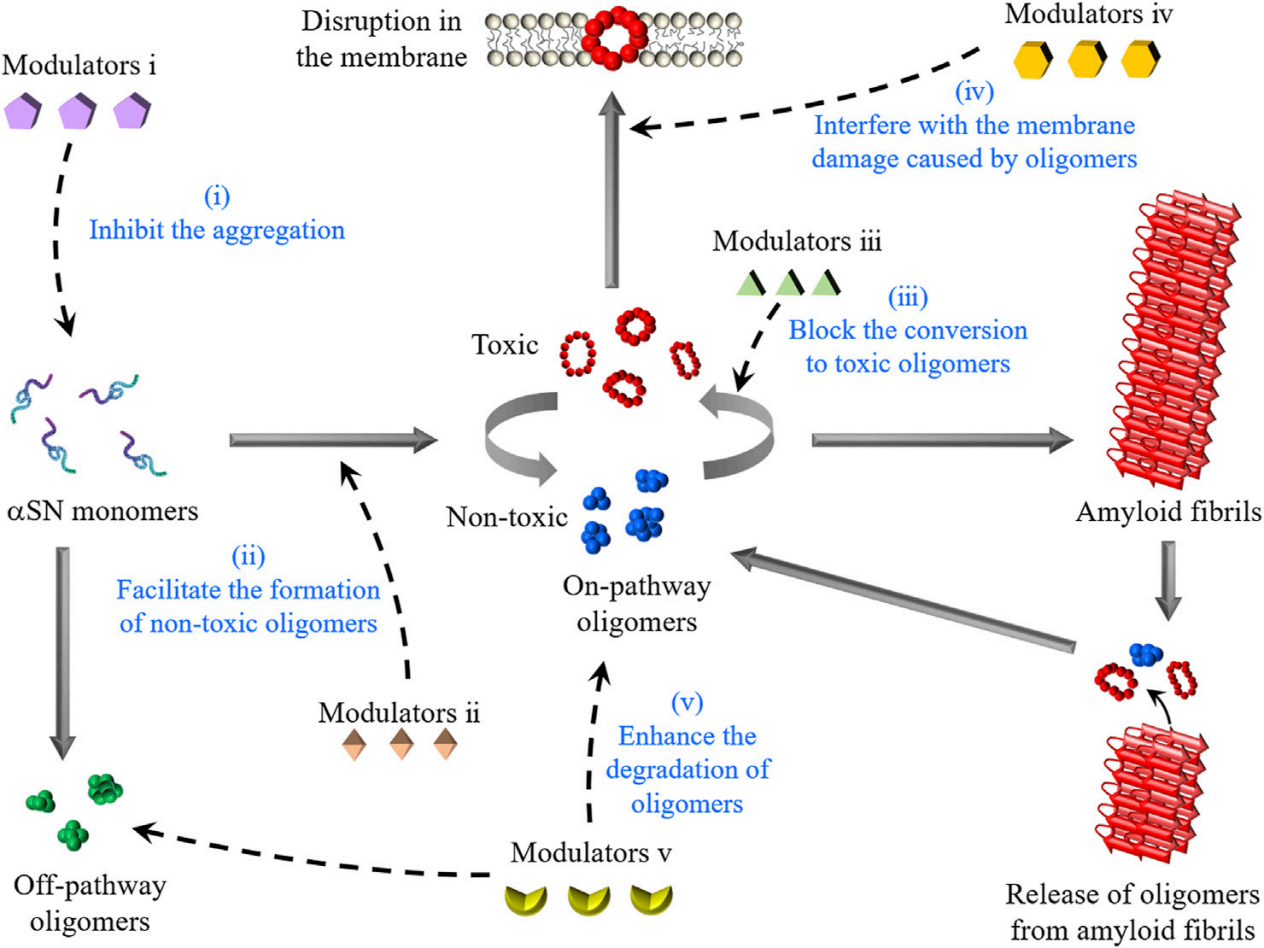
Schematic representation of the formation of αSN oligomers and intervention strategies. αSN oligomers are generated through the aggregation of monomers or the disaggregation of amyloid fibrils. The modulators described in this review interfere with the toxicity of αSN oligomers by five molecular mechanisms: (1) the inhibition of αSN amyloid formation, (2) the enhanced formation of non-toxic oligomers, (3) the stabilization of non-toxic oligomers for blocking their conversion to toxic oligomers, (4) the displacement of toxic oligomers from cell membranes, and (5) the enhancement of the degradation of oligomers.

Current PD medications in the clinic focus on mitigating major motor symptoms by administering levodopa to compensate for the dopamine deficiency-provoked disruption of the nigrostriatal pathway (Salat and Tolosa, 2013). In the hope of fundamentally altering the disease progression for a more efficacious and sustainable intervention, several studies have introduced modulators of toxic αSN oligomers, which can effectively reduce neuronal impairments and impede disease progression by multiple mechanisms (Figure 2).

#### 2.3.1 Polyphenols

Vast exploration has been made into the inhibitory functions of several polyphenols against the fibrillation of amyloidogenic proteins such as αSN, amyloid beta (A β ), tau, and prion (Freyssin et al., 2018). Common across these polyphenols manifests structural suitability conducive to inhibiting aggregation, as well as antioxidant properties underlying their efficacy. Interestingly, some polyphenolic compounds display evident specificity towards αSN oligomers. Foremost, protocatechuic acid (PCA) preferentially binds to oligomers or preformed amyloid fibrils to effectively reduce the toxicity provoked by αSN oligomers and fibrils (Hornedo-Ortega et al., 2016). Different flavonoids such as baicalein and epigallocatechin-3-gallate (EGCG) also exhibit neuroprotective features by regulating the oligomerization via several putative mechanisms. While some studies propose that baicalein prevents the formation of high-molecular weight αSN oligomers and thereby interferes with the oligomerization, other reports suggest its role in facilitating the formation of non-toxic spherical oligomers as the grounds for toxicity reduction (Zhu et al., 2004; Hong et al., 2008). Noticeably, EGCG also prevents amyloidogenesis by promoting the generation of small spherical oligomers (Ehrnhoefer et al., 2008; Zhao et al., 2017). These findings collectively highlight an important aspect of therapeutic development; stabilization of pathological oligomers into less deleterious species like spherical oligomers can likely lead to attenuated cytotoxicity with impeded disease progression. In addition, other compounds from diverse subclasses of polyphenols including curcumin, quercetin, and orcein-related molecules (O4) have been identified, highlighting their ability to modulate αSN oligomers and thereby mitigate the relevant toxic outcomes (Pandey et al., 2008; Bieschke, 2013; Zhu et al., 2013). On the other hand, different oxidization products of dopamine, namely catecholamines, are known to trigger the generation of toxic oligomers while successfully arresting the fibrillation process (Bisaglia et al., 2007; Mor et al., 2017). It is important to note that the interplay between dopamine and αSN is particularly significant under pathological conditions as αSN has a profound impact on controlling dopamine synthesis, transport, and uptake. Hence, understanding how oxidized derivatives of dopamine exacerbate the toxicity of αSN oligomers and thereby accelerate the disease progression should be underlined.

#### 2.3.2 Non-polyphenols

Different types of non-polyphenolic small molecules also display therapeutic effects by modulating the levels or properties of αSN oligomers. Authorized as a diuretic for several medical purposes including the reduction of intracranial pressure, mannitol plays a notable function in αSN oligomerization (Shaltiel-Karyo et al., 2013). Remarkably, while mannitol does not prevent the generation of oligomers, it blocks the formation of large oligomers and induces conformational changes from α-helices to unidentified structures. The secondary structure change presumably leads to an alternative aggregation pathway that manifests decreased neurotoxicity. Several compounds isolated from natural sources have also shown efficacy in alleviating oligomer-provoked toxicity. Squalamine, obtained from the spiny dogfish shark, is known to displace toxic oligomers from cellular membranes and thereby interfere with the membrane damage caused by oligomers (Limbocker et al., 2021). Active ingredients of Ginseng, particularly Rb1, can act to stabilize non-toxic αSN oligomers with negligible β -sheet content (Ardah et al., 2015). In addition, different epidemiological studies suggest cigarette smoking is linked to lower onset rate of PD. Interestingly, nicotine and hydroquinone, major inhalants of smoking may facilitate the formation of non-toxic spherical oligomers, leading to reduced toxicity (Ono et al., 2007). Finally, Anle138b is a lead candidate among various pyrazole compounds specifically designed to modulate αSN oligomers. By interacting with the hydrophobic binding pocket composed of several αSN, Anle138b exhibits preferential binding with oligomers without any proof of interaction with monomers (Wagner et al., 2013). This leads to Anle138b′s confirmed efficacy in different *in vitro* and *in vivo* studies, which also corroborate its ability to halt disease progression and prevent neurodegeneration. Currently, Anle138b is under phase I clinical trial.

#### 2.3.3 Proteins and Peptide-based inhibitors

A selective degradation of αSN oligomers can be achieved by the expression of carboxyl terminus of Hsp70-interacting protein (CHIP), a member of E3 ubiquitin ligase (Tetzlaff et al., 2008). Akin to Anle138b, one the most popular strategies in designing novel drug candidates incorporates the use of modified short peptides with sequence homology to αSN that either leads to an alternative pathway to generate non-toxic species or prevents the fibrillation process. The synthetic peptides predominantly correspond to the non-amyloid- β (NAC) component of αSN, with a special emphasis around the sequence 68–78 as this region provides the minimum fragment that preserves the features of pathological αSN. Among several short peptides, the T72P peptides, particularly the hexapeptide ^72^PGVTAV^77^ displayed the optimal result in preventing the fibrillation process and reducing the neuronal death provoked by αSN aggregation (Choi et al., 2011). While further evidence will provide a more compelling elucidation of the mechanism of action, the hexapeptide likely interacts with αSN monomers and small non-toxic oligomers and thereby blocks their conversion to toxic species. It should be noted that some peptides without any sequence homology to αSN have also been explored. Chemerovski-Glikman et al. demonstrated the ability of self-assembled cyclic D,L-α-peptides to bind with the NAC region and the N-terminus of αSN to promote the generation of non-toxic amorphous aggregates (Chemerovski-Glikman et al., 2016). Interestingly, β-synuclein, the 134-residue protein in the synuclein family, has shown its intrinsic ability to prevent the fibrillation of αSN by competitive binding to interfere with both the nucleation and aggregation steps (Brown et al., 2016).

## 3 Conclusion

In this minireview, the implications of polymorphic αSN oligomers with focus on the toxicity and disease progression in PD are outlined. It is important to note that different types of oligomers including Aβ, amylin (IAPP), and transthyretin (TTR) oligomers behave akin to αSN oligomers *in vitro* and follow analogous molecular pathways of aggregation (Nguyen et al., 2021). As such, understanding the intrinsic heterogeneity of amyloid oligomers provides insight into the pathoprogression of other diseases beyond PD. Particularly, as illustrated in the development of Lecanemab (BAN2401), which is currently in phase 3 clinical trials, preferential targeting of soluble oligomers stands as one of the most popular and promising approaches in the development of drugs for diseases with deleterious fibrillation of amyloidogenic proteins (Tolar et al., 2020). Even for drugs such as Aducanumab, which predominantly address insoluble Aβ plaques with only partial arrest of toxic oligomers, understanding the intrinsic polymorphism in oligomers is crucial as they act as templates which give rise to heterogeneity in mature amyloid fibrils. While the approval of Aducanumab remains controversial, its development process provides an important insight into achieving the same goal for PD patients; having a profound knowledge on the properties of αSN oligomers and their impact on disease progression is key to unlocking treatments and discover a potential cure for Parkinson’s.
